# MicroRNA and Breast Cancer: Understanding Pathogenesis, Improving Management

**DOI:** 10.3390/ncrna1010017

**Published:** 2015-04-20

**Authors:** Steven C. Eastlack, Suresh K. Alahari

**Affiliations:** Department of Biochemistry and Molecular Biology, LSU Health Science Center; 533 Bolivar Street, New Orleans, LA 70112, USA; E-Mail: seastl@lsuhsc.edu

**Keywords:** breast cancer, miRNA, serum biomarker, metastasis, cancer stem cells

## Abstract

The advent of the microRNAs in the early 1990s has proven to be a tremendously significant development within the purview of gene regulation. They participate in the regulation of a broad assembly of processes vital to proper cell function and the perturbation of these pathways following alteration of miRNA expression is strongly believed to contribute to the pathogenesis of cancer. This review provides a comprehensive overview of the miRNAs that have to date been well-characterized in the context of human breast neoplasia. Detailed discussion will center around their role in tumor initiation and progression, control of epithelial-mesenchymal transition (EMT), cancer stem cell formation, use as biomarkers in tissues and circulation, as well as their role in cancer treatment. In addition, attention will be given to topics which remain underexplored, such as miRNA control of cancer cell metabolism and the genomic/epigenetic origins underlying the preliminary disruption of miRNA expression in disease. This review will also address and attempt to resolve instances where discordant, inter-study findings have been reported (examples of which are replete in the literature) while also identifying bottlenecks hampering progress in miRNA research and other challenges that confront this fledgling but promising field of biomedical research.

## 1. Introduction

MicroRNAs are a subclass of non-coding RNAs which act as endogenous regulators of the cellular transcriptome. Since their recent discovery in 1993 [1,2] the collective sum of information related to miRNA biology has rapidly expanded; concurrently, so have the prospects of utilizing miRNA-based strategies to enhance diagnostic, prognostic, and therapeutic approaches in the management of disease. Despite their small size (mature miRNAs are ~22 nucleotides long), ongoing research increasingly reveals the importance of miRNAs in the molecular control of gene expression—a trend which is underscored by evidence indicating that the majority of mRNAs are targets of miRNA control [3]. Novel miRNAs continue to be identified, and as of June, 2014, estimates by miRBase report over 1800 miRNA loci have been discovered in humans [4]. MicroRNA are intimately involved in the control of normal cell physiology, including such processes as cell cycle progression, apoptosis, and cell development and differentiation [5]. Accordingly, the dysregulation of miRNA expression contributes to an enormous number of human diseases, perhaps the most conspicuous of which being neoplasia. Indeed, many specific miRNAs have already been linked to the pathogenesis of cancer with almost certainly many more still waiting to be discovered.

In US women, breast cancer (BC) is the leading cause of cancer morbidity and is second only to lung cancers in mortality [6]. BC is both a complex and heterogeneous group of malignancies, however, through genomic and transcriptomic analysis, it has been categorized into several discrete subgroups [7]. Given the numerous possible known etiologies of BC, it is not at all surprising that miRNAs are proving to be thoroughly entrenched in both the pathogenesis and progression of every subtype currently described. Interestingly, miRNAs even appear to display differential expression according to the specific molecular anomaly underlying the disease—a property that could be exploited by future screening tests to not only diagnosis BC, but potentially even distinguish the precise subtype and molecular profile of it as well [8]. The poor outcomes which characterize BC testify to the inadequacy of current diagnostic techniques. Consequently, BC presents an instance where advanced screening techniques are urgently needed. To this end, the recent discovery that miRNAs can be detected in the blood has piqued interest into whether such molecules could be used as a circulating biomarker, given the previous evidence which has already shown that tissue miRNA levels are dramatically altered many diseases [9]. Unfortunately, it remains unclear whether intercellular miRNA alterations parallel those seen among their extracellular counterparts. Furthermore, the origin and purpose of circulating miRNAs is still poorly understood and it is not yet obvious whether the altered levels of circulating miRNAs are an artifact of disease or a contributor to it. The hypothesis that circulating miRNAs serve as a means of intercellular communication is an intriguing theory accounting for this phenomenon, and has achieved widespread acceptance, though recently has been drawn into question [10].

The continued unearthing of miRNA’s role in pathophysiology will undoubtedly prove to be invaluable in improving the management of disease, where potential applications range from diagnosis and prevention, to prognosis and treatment. The diverse array of possible uses makes miRNAs unique and attractive options in the effort to reduce cancer morbidity and mortality. In this review, we provide an inclusive assessment of the current state of miRNA research as it pertains to BC. In short, we will address how miRNAs partake in the pathobiology of BC, how they might ultimately be used in its diagnosis and management, and how the current obstacles facing miRNA research are limiting more rapid advancement towards clinical application.

## 2. Function and Biosynthesis of MicroRNA

**Figure 1:**
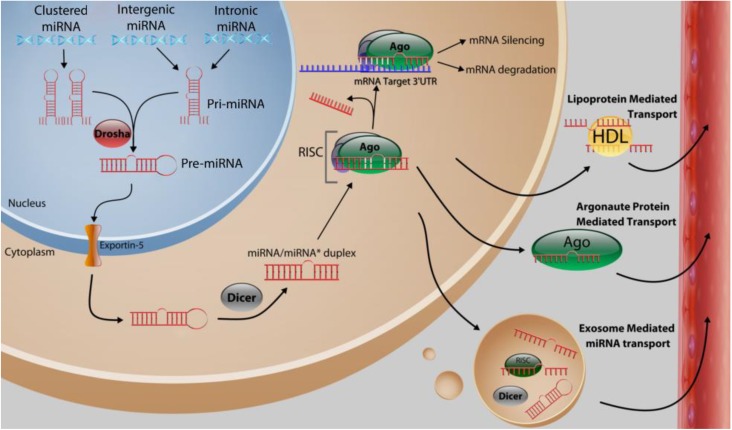
Biosynthesis of miRNAs and proposed forms of miRNA transportation within the circulation. Following transcription in the nucleus from their polycistronic, intergenic, or intronic origins, pri-miRNAs are processed by Drosha into pre-miRNAs, and transported out of the nucleus and into the cytoplasm. Here, they undergo a second processing step by Dicer into mature duplexed miRNA, and subsequently associate with the RNA-Induced Silencing Complex (RISC), which suppresses translation of specific targets according to the guidance of the miRNA within the complex. Alternatively, miRNAs may instead enter the circulation in one of several possible manners, including within lipoproteins, bound to RNA binding proteins (such as Argonaute proteins), or within exosomes.

In humans, the genomic origins of miRNAs are diverse; most commonly they arise from non-coding units with one or more miRNAs under the control of a single promoter. They can be located in either introns and exons and are found in both coding and non-coding transcriptional units [11]. Wherever their origin, they are transcribed by RNA polymerase II in the nucleus, generating “pri-miRNA,” a partially self-complementary oligonucleotide strand comprised of a core dsRNA stem-loop region flanked by two single-stranded tails (Figure 1). This precursor is subsequently cleaved by the RNase III enzyme (also called Drosha) into “pre-miRNAs,” a shorter (~70 nucleotides in length) hairpin-shaped dsRNA. This transient precursor exits the nucleus after complexing with Exportin-5, which facilitates its transport into the cytosol [12]. Here, it is further processed by Dicer, an endoribonuclease which severs the loop from the stem, forming a mature (but still double stranded) miRNA duplex. To exert its effect, a mature miRNA depends on the assembly of ribonucleoprotein complexes, referred to as the RNA-Induced Silencing Complexes (RISCs). The RISC facilitates the dissociation of passenger and guide (targeting) strands, allowing the guide strand to inhibit translation by hybridizing to a target mRNA 3’-UTR via partially complementary base pairing. The degree of complementarity appears to be an important predictor of the resulting fate of the mRNA target, as the RISC may instigate either degradation of the mRNA target (if match is highly complementary) or merely blockade translation (if the match is less highly complementary). In animals, where miRNA:mRNA complementarity is largely imperfect, this later mechanism is far more common [13]. This distinctive approach to gene expression is unique to miRNAs and allows them to “fine tune” expression to a precise level. In this role, miRNAs have ushered in a new paradigm to our understanding of how gene expression is regulated [14].

In the context of cancer biology, miRNAs are often placed within a simple classification scheme as either an oncogenic miRNA (frequently abbreviated as “oncomir”) or as a tumor suppressor miRNA (summarized in Table 1) Dysregulation of miRNAs can result in either increased or decreased expression depending on the scenario. The alteration of normal miRNA gene product expression is vulnerable to the same set of controls as are protein coding genes. In this way, both reduced and enhanced miRNA expression can promote tumorigenesis if the miRNAs subject to such events are tumor suppressors and oncomirs respectively. The similarity between oncomirs and traditional oncogenes is bolstered by the observation that they often are dysregulated by the very same mechanisms. For example, it has long been known that the majority of miRNAs are located within fragile chromosomal regions and thus are predisposed to amplification, deletion or translocation [15]. Other suspect mechanisms underlying miRNA dysregulation include epigenetic alterations, histone modification, downregulation of miRNA biosynthetic enzymes, or even alternative splicing events [16].

## 3. MicroRNA Control of Tumor Initiation

### 3.1. Let-7 MicroRNA Family

**Figure 2:**
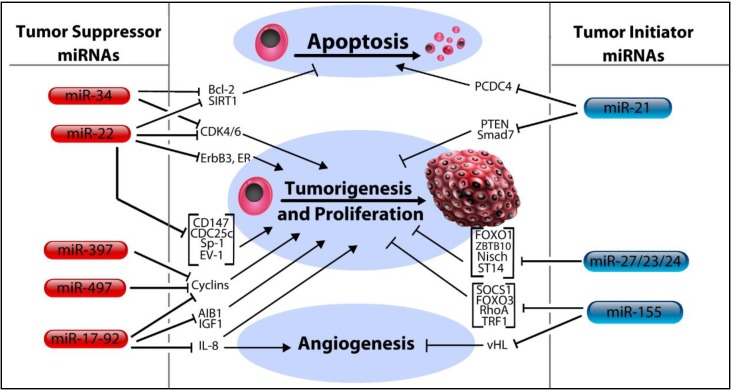
MicroRNA regulation of apoptosis, tumoriogenesis, and angiogenesis in breast cancer. MicroRNAs listed in the left column act to repress pro-cancer phenotypes by direct targeting of oncogenic mRNAs. By targeting Bcl-2 and SIRT1, both miR-22 and miR-34 promote apoptosis of cancer cells. Both these miRNAs also inhibit tumorigenesis by targeting CDK4 and CDK6. MiR-22 further inhibits tumorigenesis and cell growth by targeting ERα, ErbB3, CD147, Cdc25c, Sp-1 and Ev-1. MiR-397, miR-497, and the miR-17-92 family all act by targeting cyclin proteins. The miR-17-92 family furthermore acts by targeting AIB1, IGF1, and IL-8. Alternatively, the miRNAs in the right column target mRNAs coding for genes with known tumor suppressive functions and thus promote the development of cancer traits by cells. In this way, differing miRNAs can control tumorigenesis, cell proliferation, and angiogenesis. MiR-21 inhibits apoptosis by inhibiting PCDC4 and promotes tumor initiation by targeting PTEN and Smad7. Similarly, the miR-27/23/24 family promotes tumor initiation by targeting FOXO1, ZBTB10, ST14, and Nischarin. MiR-155 promotes tumor initiation by targeting SOCS1, FOXO3, RhoA, and TRF1, and additionally, promotes angiogenesis by targeting von Hippel Lindau (vHL) tumor suppressive factor.

In general, tumor suppressor miRNAs act by silencing genes which would otherwise enhance tumor pathogenicity. Therefore, pathology occurs when expression of these miRNAs is curtailed (miRNAs which function primarily in the control of tumoriogenesis are illustrated in Figure 2). An example of a well characterized miRNA family of tumor suppressors is the let-7 family. In humans, this family is comprised of 13 members dispersed throughout the genome in multiple polycistronic clusters, including the let-7a-1 cluster, let-7f-1 cluster, let-7d cluster, let-7a-3 cluster, and let-7b cluster [17]. Along with lin-4, let-7 bears the distinction of being the first miRNA to be discovered. Thus, its seemingly incongruous name (an abbreviation for *lethal-7*, the title of its coding gene) is an artifact of being named prior to a conventional miRNA naming scheme (although it has been given a systematic name–miR-98–the use of let-7 persists). It was discovered in the roundworm, *Caenorhabditis elegans*, in a pioneering study, assessing its role in the contextual framework of cell differentiation and stem cell regulation [18]. The authors concluded that let-7 negatively regulates gene expression by antisense base-pairing with target 3’-UTRs present in a group of heterochronic genes. Later on, it was shown to target members of the Ras family of GTPases and HMGA2, which incidentally, are both pro-oncogenes [19]. Additional findings have suggested that let-7 is dysregulated in many cancers (including breast), permitting self-renewal and tumorigenicity to proceed unchecked in its absence [20]. Let-7 has also been found to be a target of lin-28, an RNA binding protein which inhibits let-7 biogenesis, the downstream effects of which lead to the promotion of epithelial-mesenchymal transition (EMT) and enhanced self-renewal [21]. Building on this finding, the lin-28 mediated suppression of let-7 was found to be transactivated by the Wnt/β-catenin signaling cascade, providing a mechanistic link between Wnt/β-catenin signaling and the stem cell expansion correlated with loss of let-7 [22]. Let-7 also inhibits cell motility, a finding explained by its putative targeting of various actin regulatory proteins [23]. As of late, an intriguing relationship between let-7a and BRCA tumor suppressors was reported, revealing that let-7 (and miR-335) is consistently downregulated in cancers with BRCA mutations [24]. In light of all that has now been discovered concerning let-7, it is worth mentioning the results of a 2012 systematic review, wherein the authors concluded that loss of let-7 is more frequently and significantly associated with poor cancer outcomes than any other miRNA, attesting to the critical importance of the let-7 family in the preservation of normal cell physiology [25].

### 3.2. MicroRNA-17-92 Cluster

MicroRNA-17-5p (also known as miR-91) is likely the most well studied member of the miR-17-92 cluster. Initial findings suggested that it targets the AIB1oncogene (amplified in breast cancer 1) and Insulin-like Growth Factor −1, as well as documenting its ability to inhibit anchorage-independent growth [26]. A subsequent report added additional targets, including IL-8 and CyclinD, substantiating miR-17-5p’s preliminary categorization as a tumor suppressor [27]. Furthermore, in a systematic analysis of metastasis associated genes in basal-like BC, miR-17-5p stood out as a potent metastatic suppressor [28]. However, in apparent contrast to this uniformity, others have called into question whether miR-17-5p is invariably a tumor suppressor, citing evidence that it promotes tumor progression. For example, one study identified HBP-1 as a miR-17-5p target, the loss of which resulted in enhanced cell migration and invasion [29]. Another presents the curious findings that miR-17-5p simultaneously both inhibits and promotes cell proliferation, resulting from miR-17-5p targeting of over 20 genes involved in G1/S transition regulation [30]. Whatever the case, it bears mentioning that miR-27-5p is merely one of several miRNAs which has been consigned opposing roles (such is the case for miR-22, the miR-23cluster, and miR-200 as well). While the issue remains largely unaddressed, reasonable explanations do exist. For example, the cell context in which a miRNA is studied can account for radical differences in the way miRNAs behave. An excellent example of this phenomenon can be illustrated by considering the case of miR-93, a miRNA chiefly known as a regulator of cancer stem cell development. Initially, Liu *et al.* presented evidence connecting overexpression of miR-93 with induction of EMT and depletion of CSC populations in SUM159 cells [31]. However, in a subsequent study by the same group, ectopic expression of the very same miRNA actually expanded the CSC populations within MCF7 cell cultures [32]. While the alternate findings seen in the case miR-93 may be adequately accounted for by considering cell context, many more confounding reports persist without such explanations, representing a dilemma which future research will need to rectify.

### 3.3. MicroRNA-34 Family, miR-379 and miR-497

The miR-34 family, along with miR-379 and miR-497 are alike in that they share a common affinity for targeting cyclin proteins (though not exclusively). For example, miR-34c exerts control over the cell cycle by targeting CyclinD1, as well as two kinases, CDK4 and CDK6, all of which serve as vital regulators of the G2/M transition [33]. Other studies have determined that miR-34 acts to prevent cell survival following DNA damage, specifically via upregulating p53 post- irradiation *in vivo* [34]. In addition, the targeting of SIRT1 (silent information regulator 1) by miR-34 appears to rescue p53 expression, providing an additional link between miR-34 and p53 [35]. SIRT1 normally functions to suppress p53, thus, by targeting SIRT1, miR-34 safeguards expression of p53 and thus its downstream targets. The authors also report that the Bcl-2 proto-oncogene is a probable target of miR-34 suppression, showing evidence that miR-34 abrogates ectopically expressed Bcl-2’s capacity to induce proliferation and migration in BC. In addition to its role as an inhibitor of tumor initiation, others have proposed a function for miR-34 in the restraint of malignant progression as well, showing that miR-34 targets ZNF281, a zinc finger protein known to be overexpressed in cells undergoing EMT [36]. For their part, miR-379 and miR-497 primarily act as inhibitors of cell division, mainly through inhibition of cyclin proteins. A 2013 study by Khan *et al.* uncovered this role for miR-379, a previously uncharacterized miRNA in BC, demonstrating that it directly targets CyclinB1 in breast epithelial cells [37]. It has additionally been implicated as a regulator of IL-11 expression [38]. As seen with miR-379, miR-497 acts by targeting cyclin proteins, including both CyclinE1 and CyclinD1 [39,40].

### 3.4. MicroRNA-22

The case of miR-22 offers yet another instance of different investigators assigning opposing functions to a miRNA. Evidence suggesting that miR-22 targets the oncogenes ERBB3, CDC25C and EVI-1 in metastatic BC cell lines led Patel *et al.* to make the case that miR-22 behaves chiefly as a tumor suppressor [41]. These findings are in line with an earlier report that validated miR-22 targeting of the ERα receptor and noted that suppression of miR-22 is positively correlated with cancer cell growth [42]. Furthermore, miR-22 displays a distinct ability to induce cellular senescence by direct targeting of CDK6, SIRT1, and Sp1 as well as CD147 [43,44]. Notwithstanding such findings, a 2013 publication asserted quite oppositely that miR-22 actually *promotes* EMT and stem cell traits as well as enhancing aggressive metastatic disease, findings which the authors justify with evidence that miR-22 directly targets the TET family of DNA demethylases [45]. The consequence of reduced TET expression leads to the suppression of the anti-metastatic miR-200 family following hypermethylation of the miR-200 promoter. In this way, miR-22 appears to enhance metastasis through epigenetic control over tumor suppressor gene expression. In a subsequent paper, the authors report subsidiary findings to this assertion, adding that miR-22 acts as “a very potent proto-oncogene,” showing that antagonization of miR-22 reduces cancer cell phenotypes, thus reinforcing their prior conclusions [46]. Unfortunately, the dichotomous roles attributed to miR-22 remain an unsolved issue.

### 3.5. MicroRNA-21

Of all the miRNAs known to be associated with BC, miR-21 is among the most commonly upregulated, thus, it currently looks to be among the most promising examples of a miRNA biomarker for both diagnosis and prognosis of BC [47]. Functionally, numerous targets of miR-21 are known, and include genes which prevent tumorigenesis, cell invasion, and metastasis. The pro-apoptotic protein Programed Cell Death 4 (PCDC4) is one such target, furnishing miR-21 with a reputation as a potent anti-apoptotic miRNA [48,49]. Its role as a promoter of tumorigenesis, anchorage-independent growth and cell invasion stems from evidence that it targets the PTEN tumor suppressor, TPM1 and Matrix Metalloproteinase 3 (MMP3) [50,51,52]. Additionally, the recent discovery that certain MMPs may play a protective role in the process of invasion and metastasis further characterizes miR-21 as an oncogene, given that increased expression of the tumor suppressive MMP-8 seem to downregulate miR-21 expression, providing a plausible mechanism by which these tumor-defying MMPs might work [53]. Interestingly, miR-21 has even been connected to the rare breast phyllodes tumor by inducing myofibroblast differentiation via regulation of PTEN and Smad7 leading to enhanced proliferation and migration of these cells [54].

### 3.6. MicroRNA-23/27/24 Cluster

The miR-23/27/24 miRNAs are a family that promotes both tumoriogenesis and malignant progression [55]. The group is composed of two highly conserved but individually regulated clusters. For example, the homologs miR-27a and miR-27b differ only by a single nucleotide and both function as oncogenes [56]. Studies investigating the targets of miR-27a have provided the tumor suppressor FOXO1 as well as ZBTB10, the inhibition of which leads to increased expression the Specificity Proteins, a family of transcription factors associated with angiogenesis and proliferation of breast tumors [57]. In a similar fashion, miR-27b can also act as an oncomir, targeting the ST14 (suppressor of tumorigenicity 14) [58]. Both miR-27b and miR-23b have been shown to be pro-oncogenic factors in a report published by our lab, wherein both were found to be highly upregulated in BC cells in addition to decreasing the expression of the tumor suppressor protein Nischarin [59]. Additionally, expression of these two oncomirs was enhanced by Her2, EGF and TNF-α signaling via the Akt-NF-κB signaling cascade. In contrast, others have reported that miR-27b can behave as a tumor suppressor, citing findings that it decreases expression of CYP1B1, a cytochrome P450 involved with the production of procarcinogens [60]. The most recent published findings regarding this family describe a novel role as a contributor to cancer metastasis, specifically by targeting Prosaposin, a secretory protein which is inversely correlated with progression to metastasis [61].

### 3.7. MicroRNA-155

MiR-155 is an oncogenic miRNA most commonly associated with B cell lymphomas although it has been associated with an assortment of other tumor types, including breast. Notably, King *et al.* have offered findings outlining a distinct proangiogenic role for this miRNA, owing to its confirmed targeting of the von Hippel-Lindau tumor suppressor protein [62]. It furthermore has been shown to regulate cell growth and migration, invasion, EMT and apoptosis. The role for miR-155 as an oncomir can be credited to multiple studies which have revealed it to be a prolific inhibitor of tumor suppressor genes, including SOCS1 FOXO3a, RhoA, TP53INP1 and TRF1 [63,64,65,66,67]. In addition, the consistent over-expression of miR-155 makes it a viable prospect for use as a biomarker for cancer detection [68]. Perhaps most noteworthy, however, is a 2014 study in which a distinctive role for miR-155 in radioprotection was first described. The authors reveal evidence confirming that miR-155 inhibits DNA homologous recombination repair by targeting the RAD51 recombinase, thus limiting dsDNA break repair following irradiation [69]. Theoretically, knowledge of a tumor’s miR-155 status could be used to advise future clinicians concerning the utility of irradiation therapy.

## 4. MicroRNA Control of Tumor Progression, Metastasis

### 4.1. MicroRNA-206, miR-126, and miR-335

**Figure 3:**
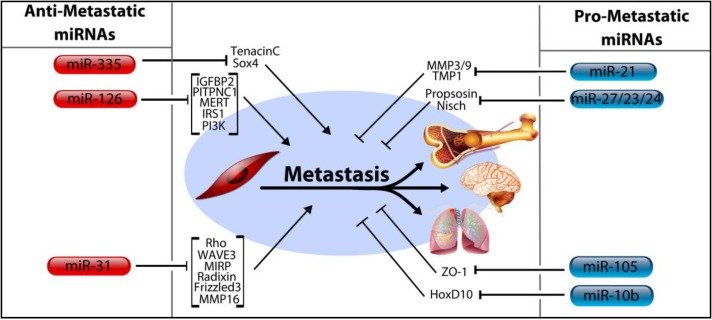
Regulation of metastasis by miRNAs in breast cancer. MicroRNAs have been found to both promote and inhibit metastasis in breast cancer by controlling the expression of numerous mRNA targets which regulate the ability of tumors to metastasize. MicroRNAs in the left column have been associated with reduced rates of metastasis, while those in the right column promote metastasis. MiR-126 has been shown to suppress metastasis and cell proliferation by targeting IGFBP2, PITPNC1, MERT, IRS1, and PI3K. Similarly, miR-335 suppresses metastasis by targeting TenacinC and Sox4. MiR-31, which also suppresses metastasis, does so by targeting its own list of pro-metastatic oncogenes, including Rho, WAVE3, MIRP, Radixin, Frizzled3 and MMP16. Pro-metastatic miRNAs include miR-21, which targets MMP3/9 and TMP1, miR-105 which targets ZO-1, miR-10b which targets HoxD10 and the miR-23/27/24 family which targets both the Propsosin and Nischarin tumor suppressors.

Complications of metastasis are the leading cause of cancer deaths [70]. Thus, miRNAs which regulate metastatic progression are the subject of intense research interest (Figure 3). MicroRNA-206 (a noteworthy member of the miR-200 family—discussed fully in subsequent sections) is typically categorized as a deterrent of cancer metastasis. This attribute was first bestowed upon miR-206 following a comparative analysis by Tavozie *et al.* in which the MDA-MB-231 BC cell line was contrasted with its two derivative metastatic cells lines: LM2 cell line (breast metastasis to lung) and BoM1 cell line (breast metastasis to bone) [71]. The comparison revealed derivative cells with depleted miR-206 expression, whereas miR-206 expression remained intact within primary tumor cells—signifying the possible role for this miRNA as a negative regulator of metastasis. The same study also found that, much like miR-206, both miR-126 and miR-335 also demonstrate a strongly repressive influence on metastasis. The depletion of both miR-335 and miR-126 is associated with poor outcomes stemming from the reduced rate of metastasis free survival observed in their absence. Corroborating these findings, the authors add that expression of these two miRNAs is largely absent in the majority of primary breast tumors. Building on this, a 2014 report supplied evidence which could account for the deficit of miR-126 in BC, citing promoter methylation of the host gene as the cause [72]. The most recent appraisal of miR-126 targets in BC includes multiple oncogenic factors such as IRS1, VEGF, PI3K, IGFBP2, PITPNC1, and MERTK [73,74,75]. Another inhibitor of tumor progression, miR-335, is able to prevent metastatic relapse by repression of the SOX4 transcription factor as well as tenascinC, a component of the ECM [71]. It also appears that miR-335 is yet another miRNA with links to BRCA activity in light of evidence that overexpression of miR-335 leads to enhancement of BRCA1 expression [76].

### 4.2. MicroRNA-31

MiR-31 is thought to be a prolific suppressor of metastasis, a finding explained by its confirmed targeting of a plethora of pro-metastatic genes. The current library of miR-31 targets includes MIRP, MMP16, Radixin, Frizzled3, Rho, and WAVE3 as well as multiple integrins; all of which seem to enhance metastasis [77,78,79]. Notably, miR-31 was also the subject of a recent investigation by Chan, *et al.* which evaluated the existence of discrete miRNA isoforms, the production of which ostensibly follows imprecise cleavage by Drosha or Dicer during miRNA processing [80]. This study represents an example of the intriguing, though poorly understood, phenomenon of differential miRNA processing. It remains unclear if isoforms of all miRNAs exist, and furthermore if such mechanisms even contribute significantly to gene expression.

### 4.3. MicroRNA-10b, miRNA-105, and the miRNA-191/425 Cluster

MiR-10b expression is significantly increased in metastatic BC cells and has been shown to be a positive regulator of both migration and metastasis [81]. Building on this identity, therapeutic silencing of miR-10b with antagomirs *in vivo* resulted in increased levels of HoxD10, a miR-10b target, leading to marked reduction of lung metastases [82]. The current body of work regarding miR-105 implies it acts primarily as an oncomir. For example, it has been shown to target the tight junction protein Zona Occludans-1 (ZO-1) leading to destruction of natural barriers in between endothelial cells, facilitating metastasis [83]. Additionally, the authors show that miR-105 is characteristically secreted by the tumor cells into the circulation prior to metastasis, suggesting a potential role as a biomarker for early stage, pre-metastatic tumors. It has also been shown to regulate metastasis and cell polarity in an indirect manner by first suppressing tristetraprolin, a regulator of epithelial polarity and metastasis [84]. Lastly, it likely contributes to epigenetic regulation by targeting DNA methyltransferases, such as DNMT3A, DNMT3B, and ZFP36 [85].

## 5. MicroRNA Control of Cancer Stem Cells

**Figure 4:**
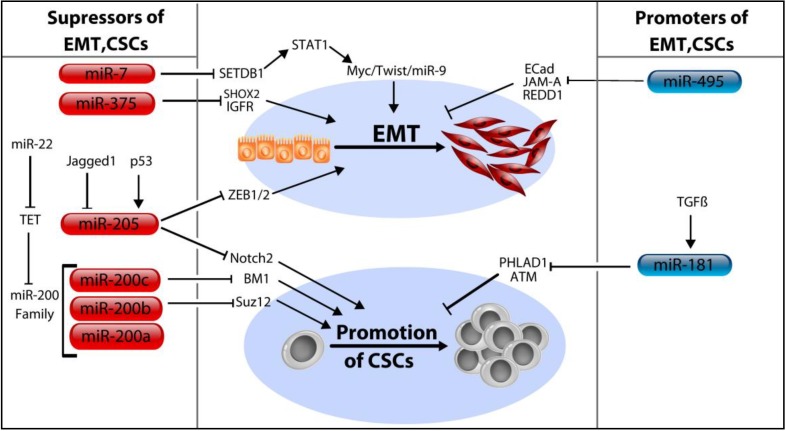
MicroRNAs regulate EMT and cancer stem cell (CSC) development in breast cancer. Both EMT and the development and promotion of CSCs are controlled by miRNAs in BC. MiR-7 is a suppressor of EMT and seems to act by first targeting SETDB1, which leads to a reduction in STAT1 signaling, and thus Myc, Twist and miR-9 signaling as well. By targeting SHOX2 and IGFR, miR-375 also leads to suppression of EMT. MiR-205 acts to suppress EMT as well as CSCs by targeting ZEB1/2 and Notch2. Its role in regulating these processes is controlled by the p53 tumor suppressor, as well as an extracellular ligand called Jagged1. MiR-200b and miR-200c prevent CSC development as well, specifically by targeting BM1 and Suz12 respectively. Oppositely, miR-495 will promote EMT by targeting three known EMT inhibitors, E-Cadherin, JAM-A, and REDD1. TGF-β appears to exert its effect over EMT by enhancing expression of miR-181, which then targets PHLAD1 and ATM.

Cancer Stem Cells (CSCs) are a subset of the tumor cell population which have acquired a phenotype reminiscent of somatic stem cells—that is, they demonstrate properties of “stemness,” including continuous self-renewal, the aptitude to mature into any cell type present in the global tumor cell population, and the proliferative capacity necessary to perpetuate the expansion of tumor cells indefinitely [86]. Because the features of CSCs are comparable to those of normal progenitor cells, it is generally believed that CSCs acquire such traits by misappropriation of normal stem cell circuits, leading to tumoriogenesis, cancer progression, and relapse. In epithelial cancers, including those of the breast, CSCs exhibit features reminiscent of epithelial cells which have undergone a morphologic transition into a mesenchyme, a process referred to as the epithelial-mesenchymal transition (EMT). These features provide rationale for the correlation between enlarged CSC populations and heightened aggressiveness of tumors [87]. Although the EMT has historically been regarded as a step within the larger process of a tumor’s advancement toward metastasis, more recent evidence is expanding the possible contribution of the EMT in cancer well beyond a solitary role as a stepping stone for invasion and metastasis. For example, preliminary evidence indicating that the EMT may bear responsibility for the generation of CSCs was published in 2008 [88]. Given the likelihood that EMT is a prerequisite for multiple aggressive cancer traits, including chemoresistance, genesis of CSCs, and metastasis, the importance of miRNA control of EMT can hardly be exaggerated (Figure 4)

### 5.1. The MicroRNA-200 Family (miR-200a/b/c, miR-429, and miR-141)

Foremost among the miRNAs known to control EMT are the miR-200 family and miR-205, which function as inhibitors of EMT and promoters of the reverse sequence (the so-called “MET”). Despite a clear propensity to suppress the EMT, the relationship between these miRNAs and control of metastasis is less apparent. Such an observation results from the ambiguity over whether EMT (and subsequent extravasation) or MET (and subsequent colonization) is the predominant event in the course of metastasis. Thus, it is feasible for a miRNA to promote EMT (and therefore CSC formation, relapse, *etc.*) and yet have little influence on metastasis. Concerning the development of mammospheres, miR-200b deters both their initiation and maintenance by targeting of Suz12 [89]. Similarly, miR-200c serves to limit stem cell renewal following confirmation that BMI1 (a protein essential for self-renewal and cell differentiation of stem cell phenotypes) is targeted by miR-200b [90]. Despite strong evidence that miR-200 inhibits EMT, one study concluded that miR-200 actually promotes metastasis, presumably due to their ability to enhance colonization at target organs [91].

### 5.2. MicroRNA-205

MiR-205 is entitled to special consideration in light of a previous analysis that suggests it is among the most significantly repressed miRNAs in breast tumors [92]. In its role as a tumor suppressor, miR-205 is involved in multiple anti-cancer processes, including suppression of cell division and invasion, tumor proliferation, and EMT [93]. Analogous to the function assigned to miRNAs upon their discovery in 1993 (*i.e.*, regulators of larval development in *C. elegans*) miR-205 regulates EMT—a transition equally as vital for embryonic development as it is for cancer progression. Specifically, miR-205 plays a critical role in regulating malignant cell behavior via targeting of ZEB1 and ZEB2, two “master regulators” of EMT [94]. Other studies corroborate its anticancer qualities, including the finding that it targets both the Her2/ErbB2 and Her3/ErbB3 growth factor receptors [95,96]. More recently discovered functions were expounded in a 2014 study in which miR-205 was found to target components of the NOTCH signaling cascade, a pathway notorious for being upregulated in invasive BC [97]. The NOTCH family constituents found to be downregulated include NOTCH2 and ZEB1 which promote maintenance of self-renewal and repression of cell polarity respectively. Importantly, this study also provides a mechanism accounting for the dysregulation of miRNA-205 in BC which has hitherto been poorly accounted for, specifically by outlining a feedback mechanism in which a tumor-stroma secreted NOTCH ligand (entitled jagged1) inhibits miR-205 though epigenetic silencing. A link between the p53 tumor suppressor and miR-205 was determined in a study seeking to characterize their role in TNBC, and concluded that p53 exerts its effect by transactivating miR-205 expression, which then functions to inhibit E2F1 and LAMC1, leading to reduced cells division and migration [98].

### 5.3. MicroRNA-7, miR-34a, and miR-375

Other miRNAs opposing EMT and the development of CSCs include miR-7 and miR-375. Zhang, *et al.* report that miR-7 inhibits EMT by targeted downregulation of SETDB1 leading to inhibition of the STAT3 pathway and ultimately, the repression of Myc, Twist, and miR-9 [99]. In addition to the multiple cell cycle regulatory proteins targeted by miR-34a (discussed above), a role for miR-34a in CSC regulation has been described as well, interestingly, by targeting CD44, the surface glycoprotein often used to differentiate stem cells from the rest of the tumor population [100]. In addition, targeting of E2F transcription factor 3 (*E2F3*), NAD-dependent deacetylase sirtuin-1 (*SIRT1*) by miR-34a were confirmed by the authors as well. Findings from a 2014 study reveal that miR-375 can limit EMT progression in BC by targeting of SHOX2 [101]. In the report, the authors outline that miR-375 levels were elevated in epithelial-like cancer cells, but conspicuously absent in mesenchymal cell types. They subsequently confirmed that the ectopic expression of SHOX2 could itself induce cells to undergo EMT. These findings echo those of a previous study published one year earlier by Ward, *et al.* in which it was also found that miR-375 repressed EMT-like properties [102]. Previous reports have also linked the dysregulation of miR-375 with disrupted of cell polarity as well as identifying IGFR1 as a target [103,104].

### 5.4. MicroRNA-181a/b and miR-495

In contrast to those above, the miR-181a/b family provides an example of a miRNAs which acts to enhance stem cell features. One study reported validation that the ATM (Ataxia Telangiectasia Mutated) tumor suppressor gene is a target, and furthermore, either the overexpression of miR-181 or depletion of ATM was sufficient to induce sphere formation in BC cells [105]. The stem cell promoting influence attributed to miR-181 was substantiated in a related study, which also added PHLDA1 to the list of miR-181 targets [106]. In addition to its CSC association, miR-181 is also involved in cell fate, invasion, migration, metastasis, and has even found use as a biomarker to both screen for cancer and assess response to treatment. The association between miR-181 and migration/invasion was established in a 2013 report by Neel and colleagues [107]. MiR-181 levels have also been found to be strongly correlated with BC metastasis, particularly in the case of TNBC, where it was highly predictive for reduced survival. In addition, they established TGF-β as directly responsible for the dramatic upregulation of miR-181 observed in BC [108]. Similarly, miR-495 also appears to enhance generation of CSCs by down regulating E-Cadherin, REDD1 and JAM-A but little else about this miRNA has been reported regarding BC [109,110].

## 6. Modification of Cancer Cell Energy Metabolism by MicroRNAs

Reprogramming of energy metabolism is an emerging hallmark of cancer cells [111]. As should now be clear from the preceding sections, the degree to which miRNAs participate in cellular functions is quite exhaustive; energy metabolism is no exception to this concept. However, the extent of miRNA control over cancer cell metabolism remains a poorly studied topic in comparison to the amount of interest surrounding the miRNA control of the processes discussed above (e.g., migration, invasion, metastasis, *etc.*). At any rate, the role miRNAs play in modifying energy metabolism in cancer cell is proving highly significant. Perhaps the most famous alteration in the metabolism of cancer cells is their enhanced utilization of glucose, a substrate which they prefer to consume anaerobically. The elevated catabolism of glucose, coupled with a reduction in oxidative phosphorylation of the glycolytic end products together comprise a phenomenon known as *Warburg effect* [112]. Evidence has now linked multiple miRNAs to control of this effect in BC. For example, miR-155 has been reported to enhance glycolysis through activation of hexokinase 2 [113]. In addition, the scope of miR-155 control over metabolism has also been extended to the tricarboxylic cycle, where it putatively exerts its effect (albeit indirectly) via enhancement of thiamine levels, a required cofactor for two TCA enzymatic complexes. Following knockdown of miR-155, two thiamine transporters SLC19A2, SLC25A19, as well as TPK1 were found to be reduced, matched by a concordant reduction in thiamine [114]. Like miR-155, miR-378-3p shows some affinity for regulating the TCA, in this case by direct targeting of ERRγ and GABPA, the loss of which results in repression of TCA component expression [115]. Enhanced glutaminolysis is another example of the metabolic reprograming seen in cancer cells and can be accounted for, at least in part, by miR-23a/b regulation, which both target mitochondrial glutaminase [116].

**Table 1:** MicroRNAs, their functions, and their targets in breast cancer.

MicroRNA	Primary Function	Target Genes	Reference
Let-7 Family	Tumor Suppressor	Ras, HMGA2	[19]
miR-17-5p	Tumor Suppressor	AIB1, IGF1, IL-8, CcnD1, HBP-1	[26,27,29]
miR-34	Tumor Suppressor	CcnD1, CDK4, CDK6, SIRT1, Bcl-2, ZNF281, CD44, E2F3, SIRT1	[33,35,36,100]
miR-379	Tumor Suppressor	CcnB1, IL-11	[37,38]
miR-497	Tumor Suppressor	CcnD1, CcnE1	[39,40]
miR-22	Tumor Suppressor	ErbB3, CDC25C, EVI-1, ERα, CDK6, SIRT1, Sp1, CD147, TET Demethylases	[41,42,43,44,45]
miR-21	Oncomir	PCDC4, PTEN, TPM1, MMP3, Smad7	[48,49,50,51,52,54]
miR-23/27	Oncomir	FOXO1, ZBTB10, ST14, Nisch, CYP1B1, PSAP	[57,58,59,60,61]
miR-155	Oncomir	VHL, SOCS1, FOXO3, RhoA, TP53INP1 TRF1, Rad51, HK2	[62,63,64,65,66,67,68,113]
miR-126	Anti-metastatic	IRS,1 VEGF, PI3K , IGFBP2, PITPNC1, MERTK	[73,74,75]
miR-335	Anti-metastatic	SOX4, TNC	[71]
miR-31	Anti-metastatic	MIRP, MMP16, Radixin, FZD3, Rho, WAVE3	[77,78,79]
miR-10b	Pro-metastatic	HoxD10	[82]
miR-105	Pro-metastatic	ZO-1, DNMT3A/B , ZFP36	[83,85]
miR-200	Inhibit EMT/CSCs	Suz12, BMI1	[89,90]
miR-205	Inhibit EMT/CSCs	Zeb1/2, ErbB2, ErbB3 Notch2	[94,95,96,97]
miR-7	Inhibit EMT/CSCs	SETDB1	[99]
miR-375	Inhibit EMT/CSCs	SHOX2, IGFR1	[101,103,104]
miR-181a/b	Enhance EMT/CSCs	ATM, PHLDA1	[105,106]
miR-495	Enhance EMT/CSCs	E-Cadherin, REDD1 and JAM-A	[109,110]

## 7. Circulating MicroRNAs as Biomarkers

Pioneering work into the existence of miRNAs in the blood was first reported by Mitchell *et al.* in 2008, who confirmed not only their existence, but also their possible use as a novel disease biomarker. If such biomarkers are ultimately implemented, they would represent a significant improvement upon current methods of evaluating response to treatment and disease recurrence (such as CA 15-3 and CEA), which have minimal preoperative diagnostic usefulness [117]. Ultimately, the clinical usefulness of a miRNA biomarker is dependent on the fact that circulating miRNAs are remarkably stable during a routine blood collection. The basis of this stability continues to be debated, but the consensus is generally that they circulate within the aegis of tumor-secreted exosomes, which protect cell-free miRNAs from the otherwise unavoidable degradation by the body’s ubiquitous extracellular RNases (Figure 1). Additionally, there is also evidence that they circulate by binding to RNA binding proteins such as Ago2, or even within lipoproteins [118]. In support of the exosome hypothesis, a current study found that, in addition to transporting miRNA species, exosomes retain the capacity to process miRNA precursors into mature miRNAs independently from the parent cell [119]. This capability is due to the presence of RISC components within the exosome itself, as well as other miRNA-associated proteins, which presumably would enable exosomes to mediate rapid and efficient transcriptomal silencing of the target cell. Unfortunately, the question of how and why these miRNAs initially enter the bloodstream remains an uncertainty. However, a growingly favorable hypothesis accounting for this phenomenon is that circulating miRNA serve as a means of intercellular communication [120]. While appealing, the legitimacy of this theory has recently come under fire.For example, one report, in which the investigators used a stoichiometric analysis of exosome content, concluded that the exosomal fraction of the blood contain only a small portion of the total circulating miRNA, with the average being far less than one molecule of miRNA per exosome [10]. Furthermore, although serum miRNA expression is clearly altered in BC it is not always apparent whether the change is the result of the underlying pathology or the cause of it.

Whatever their origin, a myriad of studies have been published implicating various miRNAs as diagnostic and prognostic agents for BC. For example, in the same year circulating miRNAs were discovered, Foekens *et al.* published a study suggesting that four miRNAs, miR-7, miR-128a, miR-210, and miR-516-3p could together be used to improve evaluation of BC progression, specifically in primary ER+ tumors that had not yet spread to lymph nodes [121]. Other studies have reported that miR-93 and miR-373 are capable of distinguishing metastatic from nonmetastatic BC [122,123]. In a prospective sister cohort study of 410 women, Godfrey *et al.* used a microarray to screen for 1,105 miRNAs, reporting 414 to have expression levels above the background, and 21 with significantly differential expression between the case and control cohorts. Narrowing their findings, they conclude that miR-18a, miR-181a, and miR-222 are the three most highly expressed miRNA in their patient cohort [124]. This is to be contrasted with another study which found quite oppositely that miR-181a levels were significantly *lower* in serum from patients with BC [125]. In a study of post-menopausal women who underwent BC resection, a global miRNA analysis found that three circulating miRNAs (miR-338-3p, miR-223 and miR-148a) were significantly lower in post-operative serum samples while one (miR-107) was increased [126]. Oppositely, three members of the let-7 family (let-7a,c,d), along with miR-126, miR-199a, and miR-335 are believed to exhibit decreased circulating levels in BC patient serum [127,128,129]. Importantly, it bears mentioning that in contrast to using serum (which is the blood product of choice to characterize miRNA levels in the majority of publications) the study by Hennegan *et al.* (which reports that repressed and enhanced serum levels of let-7a and miR-195 respectively correlate with BC) used whole blood as the biofluid from which they extracted their RNA samples. In defense of this practice, they contend that using whole blood results in improved RNA extraction capacity and increased RNA concentrations. Conversely, Pritchard *et al.* found that the inevitable hemolysis that occurs in samples containing cells renders the quantification and normalization of circulating miRNAs unreliable [130]. Using whole blood places the integrity of the results at risk due to the possibility of contamination by cell-derived miRNAs. As such, the results of whole blood studies should be more closely scrutinized.

It should be noted that the studies described above (and others like them) frequently report multiple miRNAs being used simultaneously to make more reliable prediction concerning BC diagnosis and prognosis. Because use of a single miRNA may lack the specificity necessary to make a reliable diagnosis alone, the utilization of a miRNA panel or multimarker is an appealing option to improve sensitivity. For example, Cuk *et al.* report that use of a multimarker had considerable discriminatory power in the detection BC and suggest that such a panel might be beneficial as a prescreening tool to complement current means of detection [131]. In their study, they validated their previous findings which proposed that miR-148b, miR-376c, miR-409-3p, and miR-801 are routinely elevated in BC patients, and augmented their previous list with three more—miR-127-3p, miR-376a, and miR-652—confirming seven total miRNAs in the their patient samples.

Although serum miRNAs have by now been extensively investigated, there is little agreement within the literature concerning which miRNA(s) might be the most practical as a biomarker for BC. The lack of consensus is the subject of a recent review by Witwer in which the author notes that the vast majority of miRNAs reported to be differentially expressed in BC are supported by only a single reference, and 25 of these miRNAs had discordant results between studies [132]. Moreover, of the 10 miRNAs for which there was support by more than one article, 9 of them were supported only by papers which came from the very same institution and even shared the some of the same authors. There are innumerable possible sources for the inconsistency seen in the current literature, however, from an experimental perspective, the greatest obstacle hampering progress towards more reliable data is the continued absence of a proven reference miRNA for use as an internal control. In the inaugural serum miRNA study published by Mitchell *et al.* in 2008, the authors attempted to resolve the dilemma by using synthetic miRNA “spike-ins” added directly into patient serum samples to normalize their results, but it remains uncertain if such an approach is the ideal experimental control. Nevertheless, in the period shortly following Mitchell’s report, a flurry of similar studies were published, many utilizing endogenous small RNAs as experimental normalizers which were unproven for use in serum-based assays (such as RNU6b and miR-16). Although, use of such controls is still commonplace, many reports are drawing attention to this potential fault in study design, but a uniform approach to normalization remains elusive.

## 8. MicroRNAs and Cancer Therapy: Agents of Drug Resistance

No sooner than the link between miRNAs and cancer was exposed, investigators began devising strategies to manipulate miRNA pathways in hopes of producing novel cancer treatments. Theoretically, nucleic acid-based drugs could work by either directly introducing an underexpressed tumor suppressor miRNA, or by antagonism of an overexpressed oncogenic miRNA by introduction of an anti-sense miRNA mimic. A current failing of many cancer therapeutics stems from their narrow latitude of activity, perhaps only impeding a single process within the cell. All too often, this is a barrier which cancer cells readily circumvent. By contrast, the domain of miRNAs is characterized by the tendency of miRNAs regulate a large number of targets. This quality makes exploitation of miRNAs pathways uniquely suitable for cancer therapy. To this end, multiple computational resources have been designed to achieve this, such as *miR-Synth,* a program allowing researchers to purposely design synthetic miRNAs capable of multi-site and multi-gene targeting to enhance potency of RNAi-based strategies [133]. Unfortunately, pharmacokinetic complications continue to hinder employment of therapeutic miRNAs, such as the absence of an efficient drug delivery system. Nonetheless, the growing awareness of miRNA’s underlying involvement in chemotherapeutic resistance has greatly enhanced the understanding of how cancer cells escape destruction, and this knowledge will serve as the basis for the eventual development of miRNA treatments.

The overexpression of growth factor and hormone receptors is a recurrent motif in BC. Thus, while miRNAs are relevant in all cancers, their prominent involvement in regulating these cell receptors imparts a particularly important role for them in BC. Many examples of miRNAs regulating surface receptor expression have already been noted above (e.g., miR-22, miR-205, miR-206, *etc.*). The observation that miRNAs act in this way carries important implications for cancer pharmacology, given that antagonizing such receptors is a bulwark of modern BC therapy. Thus, the use of miRNA-based agents to improve sensitivity to hormonal treatments is a current objective of miRNA research. For example, resistance to the aromatase inhibitor (AI) class of drugs (e.g., lestrozole) has been linked to overexpression of miR-128a, which targets the TGF-β signaling pathway [134]. Furthermore, it was also shown that inhibition of endogenous miR-128a could reestablish sensitivity to letrozole, as well as the growth inhibitory effects of the TGF-β pathway. Tamoxifen resistance is a frequent occurrence during BC progression and can develop during the course of treatment or *de novo* [135]. To this end, the ectopic expression of miR-221 and miR-222 is at least one source of resistance due to their targeting of p27, which prevents the initiation of apoptosis in response to tamoxifen treatment [136]. In a similar manner, miR-519a bestows resistance by preventing tamoxifen-induced apoptosis, a finding which identified miR-519a as novel oncomir in BC [137]. Alternatively, resistance to tamoxifen can also result from the overt elimination of ERα expression, a phenomenon which occurs in cancer cells that have developed constitutively active components of growth signaling pathways. The up-regulation of multiple miRNAs has been implicated in such events, including miR-221/222 and miR-206 [138]. Conversely, the loss of expression of miR-375 was linked with resistance to both tamoxifen and trastuzumab (a monoclonal antibody which targets the HER2/neu receptor) [102]. Similar to miR-375, reduced expression of miR-342 is also associated with increased drug resistance, specifically to tamoxifen [139]. Overexpression of miR-21 has frequently been shown to confer drug insensitivity in BC, and it’s proficiency to do so traverses multiple, unrelated classes of drugs. For example, evidence has linked miR-21 to trastuzumab resistance (an anti-HER2 antibody) in HER2 + BC, topotecan resistance (a topoisomerase inhibitor) in MCF-7 cells, as well as taxol resistance (a microtubule stabilizer) in breast carcinoma cells [54,140,141]. Given the manifold drug resistance resulting from miR-21 over-expression, direct targeting of this miRNA using antisense oligonucleotides is a prime example of how the use of miRNAs in cooperation with current treatment regiments could be used to improve response to therapy.

## 9. Conclusions

Despite their discovery less than twenty-five years ago, a tremendous amount of literature has already been published about these diverse and complex cell regulators. As the content accumulates, it becomes ever more important to incorporate it within the greater framework of breast cancer pathogenesis. Their potential looks increasingly promising and clinical application is becoming within reach. Continuing to make inroads towards their ultimate use in clinical settings should be the abiding philosophy of investigators in the field as future research progresses. It seems more likely now than ever before that miRNAs will emerge as a powerful resource to advance the diagnosis and management of breast cancer.
